# Multiple formation pathways for amino acids in the early Solar System based on carbon and nitrogen isotopes in asteroid Bennu samples

**DOI:** 10.1073/pnas.2517723123

**Published:** 2026-02-09

**Authors:** Allison A. Baczynski, Ophélie M. Mcintosh, Danielle N. Simkus, Hannah L. McLain, Jason P. Dworkin, Daniel P. Glavin, Jamie E. Elsila, Mila Matney, Christopher H. House, Katherine H. Freeman, Harold C. Connolly, Dante S. Lauretta

**Affiliations:** ^a^Department of Geosciences, Pennsylvania State University, University Park, PA 16802; ^b^Department of Physics, Catholic University of America, Washington, DC 20064; ^c^Solar System Exploration Division, NASA Goddard Space Flight Center, Greenbelt, MD 20771; ^d^Center for Research and Exploration in Space Science and Technology, NASA Goddard Space Flight Center, Greenbelt, MD 20771; ^e^Lunar and Planetary Laboratory, University of Arizona, Tucson, AZ 85721; ^f^Department of Geology, School of Earth and Environment, Rowan University, Glassboro, NJ 08028; ^g^Department of Earth and Planetary Sciences, American Museum of Natural History, New York, NY 10024

**Keywords:** stable isotopes, amino acids, OSIRIS-REx, Bennu, Murchison

## Abstract

Samples collected from the carbonaceous near-Earth asteroid Bennu and delivered to Earth by NASA’s OSIRIS-REx mission contain organic molecules relevant to prebiotic chemistry. Stable isotopic measurements of extraterrestrial soluble organic matter provide critical insights into the formation pathways and alteration histories of such molecules, which hold significance for understanding the origins of life. We leverage state-of-the-art techniques for picomolar-scale isotopic analyses of amino acids in samples of Bennu and, for comparison, the carbonaceous meteorite Murchison. We report intramolecular δ^13^C values for glycine, which have not previously been measured in extraterrestrial materials; molecular-averaged δ^13^C values for amino acids, aldehydes, and ketones; and δ^15^N values for glycine, β-alanine, and D/L-glutamic acid. Intramolecular carbon isotope patterns of glycine in Bennu contrast with those in Murchison, suggesting distinct formation pathways. We explore several formation mechanisms and hypothesize that the observed glycine in Murchison formed dominantly by a Strecker-like synthesis under aqueous conditions, whereas the glycine currently found in Bennu may have formed mainly by modified radical–radical reactions in primordial ices at the cold, outer reaches of the early Solar System and retained its isotopic values throughout accretion and multiple episodes of aqueous alteration. This hypothesis is supported by the highly ^15^N-enriched δ^15^N values in Bennu amino acids (+170 to 277‰). Differences in the δ^15^N values of D- and L-glutamic acid (Δ = 87‰) in Bennu affirm published reports of enantiomeric differences in meteoritic amino acids and challenge the assumption of isotopic uniformity between amino acid chiral pairs.

## Significance

Meteorites, asteroids, and comets can host prebiotic organic compounds produced by a range of processes occurring before, during, and after accretion of parent bodies. Stable isotopic measurements of these compounds provide insights into these processes, which link how planets and life developed. We measured stable isotope values of amino acids, aldehydes, and ketones extracted from samples of asteroid Bennu retrieved by NASA’s OSIRIS-REx mission. Carbon isotope values suggest that Bennu’s glycine formed mostly in primordial ices entrained in the early Solar System, whereas glycine in Murchison, a compositionally similar meteorite, formed under mild, aqueous conditions in a protoplanetary body. Additionally, nitrogen isotopes imply that the enantiomers (mirror-image molecules) of glutamic acid in Bennu samples experienced distinct formation or alteration conditions.

Organic matter in carbonaceous meteorites carries information about the synthetic and evolutionary histories of organic molecules in the early Solar System. It is widely hypothesized that prebiotic organic material on early Earth was, in part, delivered by carbonaceous meteorites and comets (1, 2). Amino acids, the monomers of proteins and critical components in metabolic processes, have long been of particular interest to astrobiologists studying the origins of life. The distribution, abundance, and stable isotopic compositions of amino acids in Solar System materials can offer profound insights, not only into the composition and chemistry of the molecular cloud and protosolar nebula that gave rise to our Solar System, but also the subsequent history of alteration and reprocessing events that occurred on meteoritic parent bodies (3, 4).

Amino acids may have formed by a combination of ice radiation chemistry before the accretion of chondrite parent bodies and during hydrothermal processing within such bodies (5) via multiple possible formation mechanisms (4). These diverse synthetic pathways depend on different precursors and reaction processes, thereby encoding a compound’s synthetic history in its isotopic structure. The ice radiation hypothesis is supported by the in situ detection of glycine—the simplest amino acid—in the coma of comet 67P/Churyumov-Gerasimenko by the ROSINA instrument aboard Rosetta, providing direct evidence that such prebiotic molecules can be synthesized and preserved in icy bodies formed in the outer solar nebula (6). Although subsequent episodes of hydrothermal processing may have affected their abundances and distribution (7, 8), experimental studies under mildly alkaline conditions suggest that aqueous alteration has a minimal effect on the δ^13^C values of amino acids (9). As a result, both δ^15^N values and intramolecular δ^13^C distributions in meteoritic amino acids may preserve information about the local chemical conditions of their parent bodies at the time of formation. Because organic synthesis can take place under a wide range of conditions, observed isotope signatures will reflect a mixture of molecules formed during early outer Solar System processes and subsequent inputs from later aqueous, geologic processes.

However, interpreting isotopic signatures of amino acids and other soluble organic compounds in meteorites can be complicated because they may be altered by atmospheric entry and/or contaminated by exposure to Earth’s environment. To circumvent this problem, pristine materials from the carbonaceous near-Earth asteroid (101955) Bennu were sampled and delivered to Earth by NASA’s Origins, Spectral Interpretation, Resource Identification, and Security–Regolith Explorer (OSIRIS-REx) mission (10). State-of-the-art analytical techniques, pico-CSIA (11) and GC-Orbitrap-IRMS (12), were used to determine the isotopic values of amino acids present in trace abundances (picomoles).

We report position-specific δ^13^C measurements of glycine; molecular-averaged δ^13^C values for amino acids, aldehydes, and ketones; and δ^15^N values for glycine, β-alanine, and D/L-glutamic acid. We compare our results to those from Murchison, a Mighei-type (CM) carbonaceous chondrite (13). Murchison, like Bennu, is hydrated and organic-rich and used as the comparative baseline extraterrestrial material for understanding organic abundances and diversity in Bennu. Bennu’s parent body developed in or accreted ices from a reservoir in the outer Solar System (14). The petrology and petrography of Bennu samples suggest that these ices melted to produce water-rich fluid, which extensively altered the anhydrous precursor materials in multiple episodes (15). Despite evidence of aqueous alteration in both Bennu and Murchison, the differing isotopic ratios in their amino acids reflect variations in the composition of precursors as well as distinct physicochemical environments for molecular synthesis.

## Results

Nineteen amino acids were identified in a homogenized powder of Bennu aggregate sample (OREX-800107-183; Methods) by comparing their retention times and mass spectra to a standard mixture of amino acids previously detected in meteorite samples. These compounds include glycine, β-alanine, L- and D-alanine, γ-amino-*n*-butyric acid, L- and D-β-aminoisobutyric acid, L- and D-α-amino-*n*-butyric acid, α-aminoisobutyric acid, L- and D-isovaline, L- and D-valine, δ-amino-*n*-valeric acid, L- and D-aspartic acid, and L- and D-glutamic acid. The absence of amino acids in the procedural blanks (*SI Appendix*, Fig. S1), the racemic-within-error D/L ratios (Table 1), and the higher-than-terrestrial δ^13^C and δ^15^N values (Table 1) indicate that the amino acids detected in OREX-800107-183 are extraterrestrial.

**Table 1.** δ^13^C (‰, VPDB; molecular-averaged and intramolecular), δ^15^N (‰, AIR), and D/L ratios for amino acids in Bennu (OREX-800107-183) and Murchison meteorite

Compound	D/L^*^	MA δ^13^C^†^	COOH δ^13^C^‡^	Cα δ^13^C^§^	δ^15^N^¶^
**Bennu (OREX-800107-183)**					
Glycine		+21 ± 6 (6)	+28 ± 13 (3)	+15 ± 4 (3)	+185 ± 10 (3)
β-alanine		+12 ± 6 (4)			+170 ± 4 (3)
D-glutamic acid	0.9 ± 0.1 (6)	+5 (1)			+277 ± 7 (3)
L-glutamic acid		+20 ± 5 (4)			+190 ± 32 (3)
D-aspartic acid	1.0 ± 0.1 (6)	+3 (1)			
L-aspartic acid			+4 (1)			
γ-aminobutyric acid		+17 (1)			
**Murchison**					
Glycine		+21 ± 3 (11)	−9 ± 15 (3)	+51 ± 13 (3)	+78 ± 6 (3)
β-alanine		+8 ± 4 (12)			+63 ± 4 (3)
D-glutamic acid	0.5 ± 0.1 (11)	+24 (1)			
L-glutamic acid		+17 ± 5 (4)			
γ-aminobutyric acid		+23 ± 11 (2)			

^*^D/L ratios calculated using peak areas from GC-IRMS chromatogram. Errors shown are SD; number of measurements is shown in parantheses.

^†^Errors shown are SD; number of measurements is shown in parantheses.

^‡^Errors shown are propogation errors, see Eq. **8** in *SI Appendix*; number of measurements is shown in parantheses.

^§^Errors shown are propogation errors, see Eq. **6** in *SI Appendix*; number of measurements is shown in parantheses.

^¶^Errors shown are SEM; number of measurements is shown in parantheses.

Table 1 presents the molecular-averaged δ^13^C and δ^15^N values for the amino acids present in sufficient abundances for isotope analysis, as well as the intramolecular carbon isotope data for the alpha carbon (Cα) and carboxyl carbon (COOH) in glycine, for both the Bennu and Murchison samples.

**Fig. 1.**
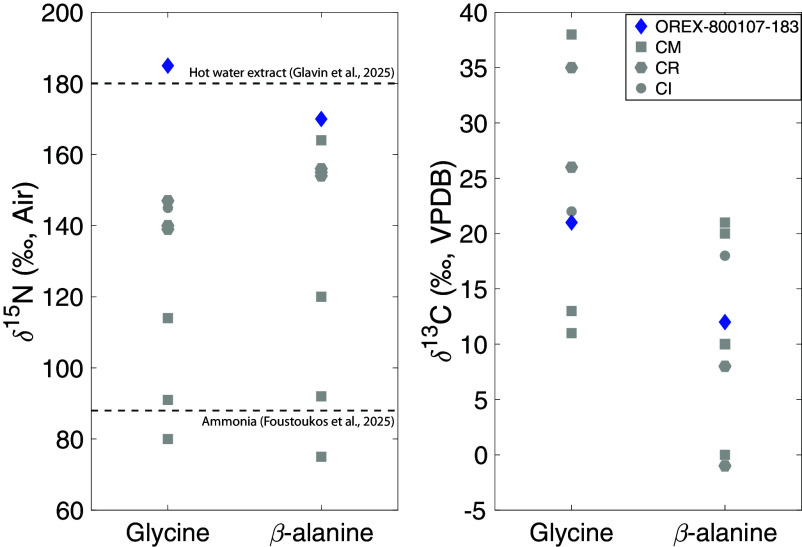
Plots of δ^15^N (‰, Air) and δ^13^C (‰, VPDB) values for glycine and β-alanine obtained in this study for Bennu (OREX-800107-183; blue diamonds), compared with literature values for CM (gray squares), CR (gray hexagons), and CI (gray circles) chondrites (4, 16, 17). The dotted lines show the δ^15^N values of a Bennu hot-water extract (14) and ammonia extracted from a homogenized aggregate of unsorted Bennu material (18).

The molecular-averaged carbon isotope values of glycine, β-alanine, γ-aminobutyric acid, and L-glutamic acid in Bennu are indistinguishable from the corresponding amino acid δ^13^C values in Murchison, within analytical uncertainties (Table 1). The molecular δ^13^C values of glycine and β-alanine in Bennu also fall within the range of previously reported values in other CM, Renazzo-type (CR), and Ivuna-type (CI) meteorites (Fig. 1) (4, 16, 17). We report the δ^13^C value of L-glutamic acid (+20 ± 5‰) in Bennu sample OREX-800107-183 with a high degree of confidence (*n* = 5), whereas D-glutamic acid (+5‰) has lower analytical confidence (*n* = 1) (*SI Appendix*).

The amino acid δ^15^N values in Bennu are higher than those in Murchison (Table 1) and other CM, CR, and CI carbonaceous chondrites (Fig. 1) (4, 16, 17). While δ^15^N values for glycine, β-alanine, and L-glutamic acid in Bennu are comparable within error, the value for D-glutamic acid is ^15^N-enriched by +87‰ compared to L-glutamic acid.

Both carbons of glycine in Bennu sample OREX-800107-183 were isotopically similar within error (Cα: +15 ± 4‰; COOH: +28 ± 13‰). In contrast, δ^13^C values for the two carbons of glycine in Murchison differed significantly: Cα was strongly enriched in ^13^C (+51 ± 13‰) relative to the COOH carbon (−9 ± 15‰).

Extract OREX-800107-127 from the homogenized Bennu aggregate sample (OREX-800107-111) contained a small suite of aldehyde and ketone compounds with abundances comparable to those in Murchison. Among these, acetone was the most abundant in both Bennu and Murchison, though their carbon isotope values differed substantially (−20.3 and +8.9‰, respectively). Table 2 presents the procedural blank–corrected abundances and molecular-averaged δ^13^C values for the aldehydes and ketones in the Bennu and Murchison samples with sufficient abundances to enable isotope analysis. All aldehydes and ketones in Bennu were relatively depleted in ^13^C, with δ^13^C values ranging from −19.3 to −1.1‰, compared to Murchison, where the values ranged from −3.4 to +17.9‰ (Table 2).

**Table 2.** Blank-subtracted abundances (nmol/g) and δ^13^C values (‰, VPDB) for carbonyls in Bennu (OREX-800107-127) and Murchison meteorite

	Bennu (OREX-800107-127)		Murchison	
Compound	Abundance^*^	δ^13^C	Abundance^*^	δ^13^C
Formaldehyde	–	n.d.	–	n.d.
Acetaldehyde	1.40 ± 0.04	(Z): −1.1 ± 3.1	1.93 ± 0.22	n.d.
Acetone	36.02 ± 0.83	−20.3 ± 2.2	14.58 ± 1.14	+8.9 ± 1.4
Propionaldehyde	0.32 ± 0.01	n.d.	0.93 ± 0.07	n.d.
2-Butanone^†^	2.43 ± 0.10	−19.3 ± 1.5	5.29 ± 0.28	−3.4 ± 1.2
Butyraldehyde	0.02 ± 0.00	n.d.	0.26 ± 0.01	n.d.
2-Pentanone	0.25 ± 0.01	n.d.	3.09 ± 0.10	(E): +17.9 ± 1.2 (Z): +14.6 ± 0.8
2-Hexanone	–	n.d.	1.51 ± 0.21	n.d.

Dashed line—not detected above blank levels.

n.d.—not determined.

^*^The carbonyl abundances represent the sum of the (E) and (Z) isomers of the PFBHA oxime derivatives, where applicable.

^†^The (E) and (Z) isomers of the 2-butanone derivatives co-eluted chromatographically.

## Discussion

### Glycine Synthesis Pathways in Murchison and Bennu.

Elements within both biotic and abiotic organic compounds carry isotope signatures that reflect the compositions of their precursor substrates as well as the imprint of isotope fractionation during synthesis (19). For extraterrestrial compounds, the contrast in isotope abundances of carbon precursors, such as carbon monoxide, aldehydes, or cyanide, can result in large isotope differences for atoms in adjacent positions (3, 20, 21). Importantly, the δ^13^C values of amino acids remain stable even after exposure to alteration in mildly alkaline aqueous fluids, suggesting they preserve the isotopic composition of their precursors and record fractionation processes associated with their synthesis (9). Thus, differences in isotope signatures indicate variations in precursor substrates, synthesis conditions, mechanisms, and associated isotope fractionations (i.e., 3, 22), rather than secondary alteration effects within parent bodies due to metasomatic reactions. To interpret isotopic patterns within glycine from the Bennu and Murchison samples, we inferred precursor carbon and nitrogen isotope signatures based on values reported in the literature or determined in accompanying compounds also present in Bennu samples (this study; 14, 23).

Proposed synthetic pathways for meteoric amino acids include Strecker-cyanohydrin synthesis (24), Michael addition to nitriles (17, 25), CO_2_ addition to amines (26), reductive amination of keto acids (27), and ammonia-involved formose-type reaction (28). Of these, Strecker synthesis is most frequently invoked as the formation mechanism for α-amino acids in meteorites (4) because the relative abundance of amino acids in some meteorites is similar to that generated by electric discharge experiments (24). During Strecker synthesis, HCN, NH_3_, and aldehydes or ketones react in the presence of liquid water, which suggests mild temperatures (~25 °C), such as those proposed for the parent bodies of both Murchison and Bennu (15, 29, 30).

Comparisons of the isotope values of HCN, NH_3_, and aldehyde or ketone precursors with both molecular-averaged and intramolecular isotope values of glycine in Bennu and Murchison samples provide constraints on whether Strecker-cyanohydrin synthesis was a likely formation mechanism. If amino acid atom positions retain isotopic signatures of their precursors under aqueous conditions (9), then the δ^15^N value of glycine should reflect that of ammonia, the δ^13^C value of the COOH carbon should represent the C moiety in HCN, and the Cα should reflect the δ^13^C value of the aldehyde source. These reactant-product isotope comparisons assume little to modest (<10‰) isotopic fractionation during formation processes.

**Fig. 2.**
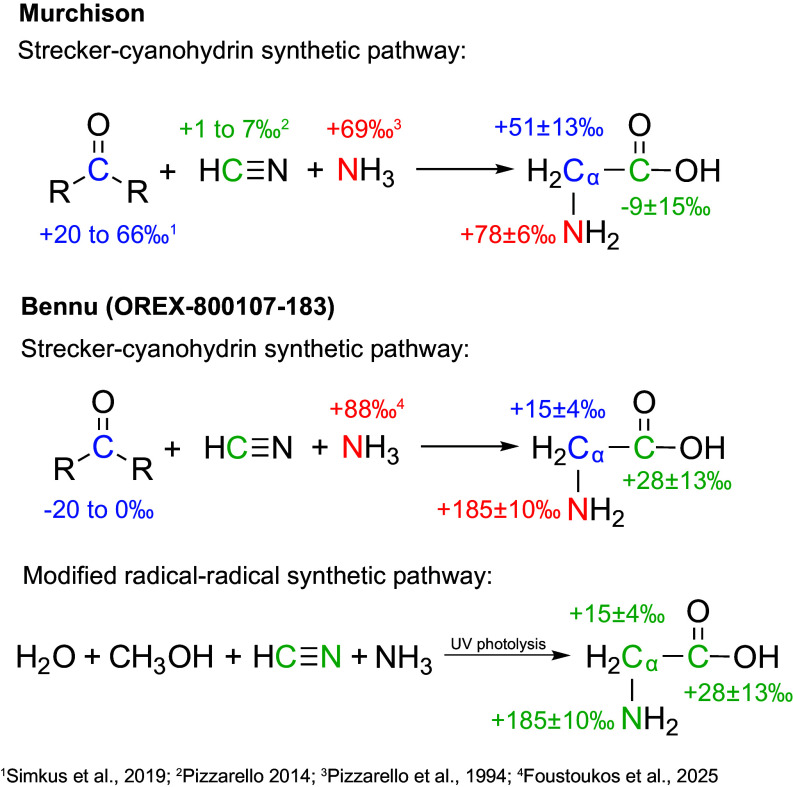
Proposed formation mechanisms for glycine for the Murchison and Bennu (OREX-800107-183) samples, with associated molecular-averaged and intramolecular δ^13^C and δ^15^N values. Literature values for the precursor compounds are from Simkus et al. (32) (1), Pizzarello (33) (2), Pizzarello et al. (31) (3), and Foustoukos et al. (18) (4).

For Murchison, the glycine δ^15^N value (+78 ± 6‰) is consistent with previous measurements of ammonia isolated from Murchison hot water extracts (+69‰) (31). Formaldehyde δ^13^C values measured in Murchison (+66 ± 3‰) (32) agree within error with the ^13^C-enriched Cα position (+51 ± 13‰). Likewise, the low δ^13^C values previously reported for Murchison HCN (+1 to 7‰) (33) overlap with the measured ^13^C-depleted COOH carbon (−9 ± 15‰). The isotopic consistency of the amine nitrogen and the Cα and COOH carbon atoms with these precursors provides strong evidence that Strecker-cyanohydrin synthesis is a plausible formation mechanism for glycine in the Murchison meteorite (Fig. 2).

Conversely, the Bennu glycine δ^15^N value (+185 ± 10‰) does not match measurements of free ammonia (~+88‰) extracted from a homogenized aggregate of unsorted Bennu material (OREX-800107-110) (18). Further, unlike in Murchison, the δ^13^C values of the Cα (+15 ± 4‰) and COOH (+28 ± 13‰) carbons of glycine in the Bennu sample we analyzed are similar within analytical uncertainty. The Cα of Bennu glycine that we measured (+15 ± 4‰) is also considerably ^13^C-enriched relative to Bennu aldehydes and ketones (−20 to 0‰; Table 2). Strecker synthesis cannot be eliminated as a potential formation mechanism because the δ^13^C value for formaldehyde, the aldehyde precursor for glycine, has not yet been measured for Bennu. If the stable carbon isotope composition for formaldehyde in Bennu samples is found to be ^13^C-enriched compared to other aldehydes, as it is in some meteorites (32, 34), Strecker synthesis could explain the observed Cα δ^13^C value. However, for Strecker synthesis to be a plausible formation mechanism for the glycine, not only would formaldehyde need to be ^13^C-enriched relative to the measured Bennu aldehydes, but it would also need to have a δ^13^C value similar to HCN. While possible, the isotopic differences between the glycine carbons and precursor compounds and between the glycine nitrogen and ammonia, and the isotope agreement between both carbon positions, suggest that Strecker-cyanohydrin synthesis was not the dominant formation pathway for the Bennu glycine that we observed (Fig. 2). This finding is similar to previous observations of aspartic acid in Murchison (21). Below, we explore alternative synthesis pathways for glycine in Bennu and assess their relative likelihood from isotopic constraints (illustrated in *SI Appendix*, Fig. S2).

Reductive amination of keto acids and ammonia-involved formose-type reaction mechanisms are both unlikely because the precursor pools represented by aldehydes, ketones (Table 2), and carboxylic acids (23) measured in Bennu samples have δ^13^C values lower than the COOH carbon in Bennu glycine. Michael addition is also eliminated from further consideration because it yields mostly β-amino acids and no α-amino acids (4).

Another possibility is CO_2_ addition to organic amines. The stable carbon isotope composition for organic amines has not yet been measured for Bennu. Future isotopic measurements of amines identified in samples of Bennu (14) will help to assess the likelihood of this formation mechanism. However, for this mechanism to explain the observed intramolecular δ^13^C values in Bennu glycine, the precursor organic amines and CO_2_ would need to have similar δ^13^C values, whereas theoretical predictions suggest that ion-molecule reactions preferentially incorporate ^13^C into the more stable ^13^CO species, leaving the CO-derived carbons such as CO_2_^13^C-enriched compared to other pools such as CH_x_ or CN (3, 4).

The modified radical–radical reaction proposed by Elsila et al. (35) is also a plausible formation mechanism (Fig. 2). This is a modification of a mechanism originally proposed by Woon (36), in which a radical–radical reaction formed glycine via the dehydrogenation of H_2_O and CH_3_OH, followed by the hydrogenation of HCN. According to the original mechanism, the Cα and N in glycine would derive from HCN and the COOH carbon from CH_3_OH. However, an isotopic labeling experiment performed by Elsila et al. (35), which examined the formation of amino acids in UV-irradiated ices containing H_2_O, CH_3_OH, HCN, and NH_3_, revealed that the major (~60%) glycine product derived both carbons from HCN. Elsila et al. (35) suggested that amino acid precursors, such as nitriles, are formed by ice photochemistry and that these precursors are then converted to amino acids by hydrolysis. Notably, the photochemical formation of glycine from interstellar ices in the outer Solar System, as proposed in this work, aligns with the suggested mechanism for the synthesis of nucleobases—particularly in explaining the elevated pyrimidine-to-purine ratio observed in Bennu relative to Murchison (14). If amino acids were formed via photochemically stable nitrile precursors (37) rather than acids, HCN would have contributed the N and both the Cα and COOH carbon positions to the amino acid product. This would produce similar intramolecular carbon isotope values, as observed for glycine in Bennu. Future carbon and nitrogen isotopic measurements of Bennu HCN are needed to test this hypothesis.

While we cannot definitively identify the formation pathway for glycine in the Bennu sample, the isotopic data presented here provide insight into which mechanisms were more likely than others. We hypothesize that the modified radical–radical reaction would be the most parsimonious explanation for the similar intramolecular δ^13^C values. Future efforts to measure isotopic data for formaldehyde, amines, and HCN will help to refine and test this prediction. Bennu’s parent body experienced geologic processes that likely influenced the abundance and distribution of organic molecules. Although He et al. (9) showed that aqueous alteration under mildly alkaline conditions had minimal impact on the δ^13^C values of amino acids, other forms of alteration are still a possibility. The complex chemical environments of Bennu’s parent body, including brine fluids during some stages, could have affected amino acid isotope values via interactions with minerals, salts, and/or alkaline fluids (as detected in Bennu samples; 38). For example, Fox et al. (39) demonstrated that the sorption of amino acids to mineral surfaces through hydrogen bonding can induce < 10‰ isotopic fractionation at the carboxyl carbon in glycine. Though modest, this fractionation should be considered if future isotopic measurements of Bennu’s HCN precursor pool allow for a comparison with our glycine carboxyl carbon δ^13^C value.

It is also possible that glycine can be synthesized in post-accretion aqueous environments or formed from the alteration of insoluble organic matter (IOM) (8, 32). Low-temperature aqueous alteration of IOM has been proposed as a possible source of organic compounds (e.g., CO, organic acids, and CO_2_) in carbonaceous chondrites (40). While the aqueous alteration of IOM could have been a source of amino acids or their precursors in Bennu’s parent body, the more labile, aliphatic moieties of IOM are expected to be relatively ^13^C-enriched relative to the aromatic portions (41), contrasting with the relatively low δ^13^C values we found for Bennu aldehydes and ketones (Table 2).

Finally, we cannot exclude the possibility that glycine formed by a yet unidentified synthetic mechanism during which isotopic fractionation was larger than assumed in this discussion (<10‰), resulting in mismatches between precursors and amino acid isotope values (3, 42). Nor can we rule out that different chemical or environmental conditions on Bennu and Murchison (or their parent bodies) resulted in different amino acid δ^13^C values despite similarities in synthetic pathways or precursor materials. However, the intramolecular δ^13^C and ^15^N-enriched nitrogen isotope values reported here suggest that the glycine that survived was dominantly formed preaccretion or from precursors retaining interstellar heritage (43). In this latter case, the currently observed aldehydes and ketones in Bennu would need to represent the isotopic values of a more recent reservoir that does not match the original primordial precursors. Regardless of the exact formation mechanisms, differences among the observed intramolecular carbon isotope values for glycine in Murchison and in Bennu suggest respective differences in the location, precursor pools, and/or mechanism of formation of indigenous amino acids.

A prevailing hypothesis postulates meteoritic amino acids formed at temperatures between 0 and 25 °C (29, 44) and in liquid water on meteoritic parent bodies (24, 25, 45, 46). Consistent with this long-held interpretation, isotopic measurements of Murchison glycine and its precursor compounds support a Strecker-like synthesis under aqueous conditions. Although we cannot rule out Strecker synthesis as a formation mechanism for glycine in Bennu (as discussed above), we suggest instead that the amino acids currently found in Bennu might have formed mainly from the energetic processing of ices containing H_2_O, CH_3_OH, HCN, and NH_3_ before the accretion of Bennu’s parent body and preserved this isotopic signature throughout multiple episodes of aqueous alteration. This aligns with a growing body of evidence that supports the formation of amino acids and nucleobases from interstellar photochemistry (35, 47, 48). Icy origins for Bennu’s amino acids are supported by observations of a highly ^15^N-enriched hot-water extract (+180 ± 47‰) (14), which match our δ^15^N measurements for glycine, as well as for β-alanine and L-glutamic acid (Table 1). The ratio of purines to pyrimidines is also much lower in the analyzed Bennu material (0.55) than in Murchison (~2.8), which suggests that Bennu’s chemical precursors were inherited from a cold molecular cloud or the outer protoplanetary disk (see ref. 14).

Differences in the location of the parent body in the protoplanetary disk or differences in temperature or length of time during which aqueous alteration occurred could have contributed to the observed isotopic differences between Bennu and Murchison. We therefore caution it is possible that both Murchison and Bennu contain a mixture of amino acids synthesized at different points in time, under different chemical conditions, and by different mechanisms. Materials derived from parent bodies formed further out in the protoplanetary disk (e.g., Bennu) show greater evidence of primordial synthesis. It is difficult to distinguish whether this represents a greater abundance of amino acids formed by primordial synthesis relative to those formed by Strecker synthesis or better preservation of primordial amino acids, possibly due to their associations within heterogeneous materials or due to lower temperatures and/or shorter periods of aqueous alteration. Untangling such molecular histories remains an active area of research.

### Nitrogen Isotope Differences for Amino Acid Enantiomers Suggest Complex Origins.

In the Bennu aggregate sample analyzed here, the δ^15^N value of D-glutamic acid (+277 ± 7‰) is 87‰ higher than L-glutamic acid (+190 ± 32‰), even though the enantiomers are racemic. Differences in the δ^15^N values between D- and L-amino acid enantiomers have been observed previously in both CM and CR chondrites. In some cases, the L-enantiomer exhibited lower δ^15^N values than the D-enantiomer—e.g., alanine (Δ = 81‰), α-aminobutyric acid (Δ = 53‰), and valine (Δ = 18‰) in GRA 95229 and alanine (Δ = 185‰) in QUE 99177 (4). While lower δ^15^N values of the L-enantiomer are typically attributed to terrestrial contamination, especially when accompanied by L- enantiomeric excess, little explanation has been offered to explain higher δ^15^N values in L-enantiomers—e.g., alanine in Murchison (Δ = 63‰) and EET 92042 (Δ = 72‰)—other than possible contributions from unidentified coeluting peaks (4). Here, we leveraged the high resolving power of the GC-Orbitrap-IRMS and single ion monitoring to isolate a single ion fragment for the amino acids of interest. This effectively limits contributions from extraneous compounds to the amino acid isotopic measurements. Further, if any such contributions to the targeted ions were sufficient to account for the observed isotopic differences, they would be readily detected in the peak shape and by the presence of other fragment ions.

We present three possible hypotheses to account for differences in the δ^15^N values of chiral amino acids, specifically in relation to the notably ^15^N-enriched D-glutamic acid in the pristine asteroidal material from Bennu: 1) distinct nitrogen reservoirs, 2) nitrogen exchange between amino acids and mineral-bound nitrogen, and 3) fractionation due to amino acid–mineral interaction. For each of these hypotheses, once an isotopic imbalance exists, the enantiomers can racemize under the cool aqueous conditions of Bennu’s parent body in geologically short times (49). While speculative in nature, it is our hope this discussion serves to foster future experimental and modeling studies that can evaluate the hypotheses presented here or generate new ones.

With the first hypothesis, it is possible that the microenvironments where amino acids formed contained distinct nitrogen isotopic compositions. Even when bulk products are racemic, at the smallest scales, D- and L-enantiomers are not necessarily in equal proportions. While HCN, amines, and ammonia—key precursors for the amino group of amino acids—have not yet been detected with δ^15^N values high enough to explain the ^15^N-enrichment observed in D-glutamic acid, the δ^15^N value of a Bennu hot-water extract (consisting of ammonia, amines, amino acids, N-heterocycles, and other N-containing molecules) is +180 ± 47‰ (14). This hot-water extract contained ~40% (~13.6 µmol/g) ammonia (14), which has a δ^15^N value of approximately +88‰ (18). Isotope mass balance dictates that the non-ammonia portion of the hot-water extract has a δ^15^N value of +241‰, a value that approaches the ^15^N-enrichment observed in D-glutamic acid. Moreover, bulk nitrogen isotope measurements of Bennu samples have revealed extremely heterogeneous values. At the nanoscale, δ^15^N values range from −558 to +3545‰ (50), suggesting the potential existence of multiple heterogenous nitrogen reservoirs.

A second hypothesis entails nitrogen exchange between amino acids and mineral-bound nitrogen. At low temperatures, ammonia can undergo proton exchange with phyllosilicates, forming ammonium-bearing clays (51), a mineral type that has been detected on the asteroid Ceres (52, 53). During adsorption–desorption processes, ammonia can become enriched in ^15^N relative to its source (54). Experimental evidence has shown that nitrogen isotope exchange can occur between organic matter and nitrogen sources such as ammonia and mineral-bound ammonium during thermal alteration (55). Furthermore, De Angelis et al. (56) reported that ammonium phyllosilicates often contain accessory minerals, including calcite, which are known to exhibit significant chiral selectivity toward D- and L-enantiomers (57). We speculate that differences in the extent of nitrogen exchange between D- and L-amino acids and a ^15^N-enriched phyllosilicate-bound ammonia in Bennu’s parent body could have contributed to the elevated δ^15^N values in D-glutamic acid. Furthermore, ammonia and ammonium can also have distinct δ^15^N values under equilibrium conditions (20, 58). Differences in these nitrogen pools combined with the nitrogen exchange mechanism described above could further exacerbate nitrogen isotopic differences.

Alternatively, there could be differential interactions between individual enantiomers and their surrounding mineral matrix. Previous studies have demonstrated that certain minerals can selectively adsorb one chiral form over the other (57), and that this sorption of amino acids to mineral surfaces can induce isotopic fractionation, particularly through hydrogen bonding (39). In their analysis of position-specific δ^13^C values, Fox et al. (39) reported isotopic fractionation sufficient to obscure biosynthetic signals, although overall, differences were generally modest (i.e., <10‰). If D-glutamic acid preferentially interacted with mineral surfaces via its amine group, fractionation due to sorption could contribute to the nitrogen isotopic differences in D- and L-glutamic acid observed in the Bennu sample we analyzed.

δ^15^N measurements of additional chiral amino acids will provide a better understanding of how common nitrogen isotopic differences are among amino acid chiral pairs in pristine asteroid samples. The initial results for glutamic acid from the Bennu aggregate sample presented here are consistent with previously reported differences in carbonaceous chondrites (4). These findings are an important caution against assuming that chiral amino acids have the same isotope values, formed from the same precursor pool, or experienced identical postformation alteration processes. Although the pristine Bennu amino acids were racemic, it is not uncommon for excess abundance of the L-enantiomer of proteinogenic amino acids to be observed in meteorite samples that have undergone prolonged exposure to the terrestrial biosphere. If future measurements of chiral amino acids in pristine asteroid samples substantiate isotopic differences between D- and L-enantiomers, the viability of using nitrogen isotopes as a test of how well D/L ratios represent potential terrestrial contributions (e.g., Table 5 in ref. 59) could be called into question.

### Conclusions.

The contrasting intramolecular δ^13^C values of glycine in Murchison and Bennu, reported here, point to distinct formation histories. These findings underscore the diversity of amino acid formation and alteration processes in extraterrestrial environments. The Murchison glycine results are consistent with Strecker synthesis in an aqueous environment within the parent body. While we currently lack a complete isotopic dataset of relevant amino acid precursors for Bennu, the isotopic agreement between both glycine carbon positions, and the disagreement between the Cα and aldehyde precursor compounds, suggest that Strecker-cyanohydrin synthesis might not be the dominant formation pathway for the Bennu glycine we measured. Instead, we hypothesize that this glycine was formed mainly by photochemical pathways and remained unaltered throughout the accretion and multiple episodes of aqueous alteration experienced by Bennu’s parent body. Additionally, a sizable difference in ^15^N enrichment observed between D- and L-glutamic acid in the analyzed Bennu sample challenges assumptions of isotopic uniformity among chiral amino acids and highlights the need for further investigation. Together, these observations demonstrate that amino acids in extraterrestrial materials originate from diverse precursor sources or formation mechanisms and/or undergo varied postformation alteration, emphasizing the importance of comprehensive isotopic analyses when assessing the origin and potential terrestrial contamination of meteoritic organic compounds.

## Materials and Methods

### Sample Extraction and Processing.

To reduce the likelihood of chemical heterogeneity, a 6.425 g aggregate sample (OREX-800107-0) consisting of fine, intermediate, and coarse particles (<0.5 cm) was crushed and homogenized in an ISO 5 HEPA-filtered laminar flow bench at NASA Johnson Space Center (60). Of this homogenized aggregate sample, 4.056 g (OREX-800107-111) was transferred to NASA Goddard Space Flight Center (GSFC) to be subsampled for organic analyses.

A 0.2697 g subsample (OREX-800107-183) of the homogenized aggregate sample and a 0.4135 g sample of the CM2 Murchison meteorite (originally provided by the Chicago Field Museum) were hot-water–extracted, acid-hydrolyzed, and desalted following published methods (14) for amino acid analysis, in parallel with a powdered silica blank, which served as a processing control, and a procedural blank at GSFC. A 2.6199 g subsample (OREX-800107-127) of the homogenized aggregate sample was hot-water–extracted, processed, and analyzed for aldehyde and ketone compound abundances and stable carbon isotope measurements at GSFC (following the protocol in refs. 32, 61). See *SI Appendix* for additional description of sample extraction and processing.

### Compound-Specific Isotopic Analysis (CSIA).

An in-house solution of 20 amino acids commonly detected in meteorites was used to identify the amino acids present in the Bennu sample and serve as a reference standard for isotopic measurements (*SI Appendix*). A separate stock solution containing only L-alanine (IsoAnalytical) with a known carbon isotope value was prepared as a working laboratory standard. Samples and standards were derivatized by methylation of the COOH group and trifluoroacetylation of the amine group, producing N,O-bis(trifluoroacetyl) methyl esters (protocol adapted from ref. 62).

Amino acid molecular-averaged δ^13^C measurements were conducted at The Pennsylvania State University using the pico-CSIA instrument design as described in Baczynski et al. (11), with the exception that amino acids were analyzed without the use of the cryogenic water trap. The pico-CSIA system employed a Trace 1310 (Thermo Fisher Scientific) gas chromatograph equipped with a programmable temperature vaporization (PTV) injector that was coupled via a GC Isolink (Thermo Fisher Scientific) and a Conflo IV to a MAT 253 isotope ratio mass spectrometer (Thermo Fisher Scientific). A custom-ordered chiral fused silica capillary column (Macherey-Nagel Lipodex E; 25 m long; 0.10 mm ID) was used to achieve enantiomeric separation. The PTV injector was held at 200 °C and operated in splitless mode. The GC carrier gas (helium) had a programmed pressure method to ensure a consistent flow of ca. 0.48 mL/min. The oven program began at a temperature of 60 °C (held for 1.5 min), was ramped to 100 °C at 25 °C/min (no hold) then to a maximum temperature of 200 °C at 10 °C/min with a final hold time of 6 min. The auxiliary gas pressure to the micro‐fluidic splitter was held at 24 psi throughout the run.

Nitrogen and intramolecular carbon isotope measurements were performed at The Pennsylvania State University on a Q-Exactive Orbitrap mass spectrometer (Thermo Scientific) with samples introduced via a modified TRACE® 1310 GC (Thermo Scientific) (12). Samples were injected into a split/splitless injector operated in splitless mode. Chromatographic separation was carried out on a 50 m × 0.25 mm ID capillary column (LIPODEX® E; Macherey-Nagel) with a 1.0 mL/min helium carrier gas flow. The GC oven temperature program was set to start at 60 °C and ramp up 2 °C/min up to 100 °C, followed by a 4 °C/min ramp up to 200 °C held for 20 min. Ions produced by electron impact (70 eV) were scanned within a 10 Da scan range centered around the mass of the unsubstituted molecular ion of each fragment. The following molecular ions were selected: *m/z* 126 Da for glycine, *m/z* 139 Da for β-alanine, and *m/z* 152 Da for D/L-glutamic acid. A nominal mass resolution (*m*/Δ*m*) of 60,000 at *m/z* 200 was used across all analyses. The Automatic Gain Control (AGC) target was set to 200,000 based on the relative abundance of the target fragments. Each fragment was analyzed three times (*n* = 3). Between each sample run, the in-house standards were measured using the same concentration and analytical conditions to account for instrument variability.

The aldehydes and ketones were derivatized using an optimized Environmental Protection Agency (EPA) Method #556 with O-(2,3,4,5,6-pentafluorobenzyl) hydroxylamine hydrochloride (PFBHA) (32) and analyzed by GC-MS coupled with IRMS at GSFC. GC separation used a 5 m base-deactivated fused silica guard column (Restek, 0.25 mm ID) and two 30 m length × 0.25 mm I.D. × 0.5 µm film thickness Rxi-5ms capillary columns (Restek) connected using SilTite μ-Union connectors (Restek). The GC oven temperature program started at an initial temperature of 60 °C, then was held for 5 min, ramped at 10 °C/min to 140 °C, ramped at 5 °C/min to 190 °C, ramped at 25 °C/min to 310 °C, and held for 6 min. The carrier gas used was ultrahigh-purity helium (5.0 grade) at a 2.5 mL/min flow rate.

Additional description of the amino acid, aldehyde, and ketone data processing methods and isotope corrections can be found in the *SI Appendix*.

## Supplementary Material

Appendix 01 (PDF)

## Data Availability

Amino Acid and Carbonyl Isotope Data have been deposited in AstroMat (https://doi.org/10.60707/0134-b672 (63); https://doi.org/10.60707/8nap-2d05 (64); https://doi.org/10.60707/gh1z-1r87 (65); https://doi.org/10.60707/40wp-e994 (66); https://doi.org/10.60707/pynf-qf21 (67).
