# Supplementary material for: Multiple formation pathways for amino acids in the early Solar System based on carbon and nitrogen isotopes in asteroid Bennu samples
Source: Proc Natl Acad Sci U S A. 2026 Feb 9;123(8):e2517723123. doi: 10.1073/pnas.2517723123 (PMC12933079; doi:10.1073/pnas.2517723123)
Supplement: Supplementary file 1 — Appendix 01 (PDF) [file pnas.2517723123.sapp.pdf]

Multiple formation pathways for amino acids in the early Solar System based on carbon and nitrogen isotopes in asteroid Bennu samples

Allison A. Baczynski[1,8*] and Ophélie M. Mcintosh[1,8*], Danielle N. Simkus[2,3,4], Hannah L. McLain[2,3,4], Jason P. Dworkin[3], Daniel P. Glavin[3], Jamie E. Elsila[3], Mila Matney[1], Christopher H. House[1], Katherine H. Freeman[1], Harold C. Connolly Jr.[5,6,7], and Dante S. Lauretta[5]

[1]Department of Geosciences, Pennsylvania State University, University Park, PA, USA
[2]Department of Physics, Catholic University of America, Washington, DC, USA
[3]Solar System Exploration Division, NASA Goddard Space Flight Center (GSFC), Greenbelt, MD, USA
[4]Center for Research and Exploration in Space Science and Technology, NASA GSFC, Greenbelt, MD, USA
[5]Lunar and Planetary Laboratory, University of Arizona, Tucson, AZ, USA
[6]Department of Geology, School of Earth and Environment, Rowan University, Glassboro, NJ, USA
[7]Department of Earth and Planetary Sciences, American Museum of Natural History, New York, NY, USA
[8]These authors contributed equally.
*Corresponding authors: Allison A. Baczynski (aab27@psu.edu) and Ophélie M. Mcintosh (omm5285@psu.edu).

Extended Methods

Amino acid extraction:

All glassware, ceramics, and tools used for sample preparation and analysis were thoroughly rinsed with Milli-Q ultrapure water (18.2 Ωcm, < 3 ppb total organic carbon) and either heated above 460°C for more than 8 hours or solvent-rinsed with methanol, dichloromethane, and hexanes to eliminate contaminants.

The Bennu sample (a homogenized powder of aggregate material; OREX-800107-183, 269.7 mg), processing control (powdered silica, 267.3 mg), and Milli-Q ultrapure water blank were flame-sealed in 5 mL glass ampoules with 1 mL of Milli-Q ultrapure water and heated at 100°C for 24 hours. After cooling to room temperature, the ampoules were centrifuged at 3000 rpm for 3 minutes to separate the supernatant, which was pipetted into 10 mm test tubes and dried under vacuum. Acid vapor hydrolysis was performed using 1 mL 6N HCl (Tamapure-AA-10 HCl, 20% concentration, metallic impurity level < 10 pg/mL) in a flame-sealed ampoule at 150°C for 3 hours. Once cooled to room temperature and dried under vacuum, samples were desalted with BIORAD AG50W-X8 (100-200 mesh, hydrogen form) cation exchange resin columns. The desalting process involved 2 M sodium hydroxide (NaOH), 1.5 N HCl, and 2 M ammonium hydroxide (NH_4OH). The 2 M NaOH solution was prepared by dissolving 32 g of anhydrous NaOH pellets (Sigma-Aldrich, >97% purity) in 400 mL of Milli-Q ultrapure water; the 2 M NH_4OH solution was prepared using ammonia gas (Air Products) and Milli-Q ultrapure water under vacuum conditions.

Amino acid derivatization:

Samples and standards were derivatized by methylation of the carboxyl group and trifluoroacetylation of the amine group, producing N,O-bis(trifluoroacetyl) methyl esters, using a protocol adapted from Corr et al. (1). 100 µL of methanol was added to each sample and standard vial and placed on ice. 25 µL of acetyl chloride was added dropwise, and vials were tightly capped and heated at 80°C for 1 hour. The methyl esters were dried under a gentle stream of nitrogen gas at room temperature. Next, 120 µL of hexanes and 60 µL TFAA were added to each vial and

samples were heated at 70°C for 30 minutes. The N, O-bis(trifluoroacetyl) methyl esters were dried under a gentle stream of nitrogen gas at room temperature prior to reconstitution in hexanes for isotopic analysis.

Amino acid standard mixture:

The in-house amino acid mixture contained α-aminoisobutyric acid (98%, Sigma Aldrich), L-isovaline (95%, AstaTech), D-isovaline (97%, AmBeed), D-alanine (≥ 98%, Merck), D-valine (≥ 98%, Sigma Aldrich), L-alanine (Arndt Schimmelmann, Indiana University), D-α-aminobutyric acid (98%, Merck), L-valine (USGS75), L-α-aminobutyric acid (≥ 99%, Merck), glycine (≥ 99%, Sigma Aldrich), D-β-aminoisobutyric acid (≥ 99%, Santa Cruz Biotechnology), L-β-aminoisobutyric acid (≥ 97%, Merck), β-alanine (99%, Sigma Aldrich), D-aspartic acid (99 %, Sigma Aldrich), L-aspartic acid (≥ 98%, Sigma Aldrich), γ-aminobutyric acid (≥ 99%, Sigma Aldrich), D-glutamic acid (≥ 99%, Sigma Aldrich), L-glutamic acid (USGS40), δ-amino-n-valeric acid (≥ 98%, TCI), and ε-amino-n-caproic acid (≥ 99%, Sigma Aldrich). 0.5 mg of each amino acid was diluted in a 4 mL 3:1 H_2O:methanol stock solution. An additional stock solution containing only L-alanine (IsoAnalytical) with a known carbon isotope value was prepared as a working laboratory standard. The amino acid mixture and L-alanine standards were derivatized in parallel with OREX-800107-183, the powdered silica blank (processing control), and the procedural blank, and they were analyzed before and after unknowns.

Amino acid pico-compound-specific isotope analysis data corrections:

Each pure amino acid standard was analyzed by elemental analysis–isotope ratio mass spectrometry (EA-IRMS). The carbon isotope value of the carbon added during derivatization (and any kinetic isotope effects associated with derivatization) was determined empirically for each individual amino acid by comparing the isotope value of the pure amino acid standard (EA-IRMS) to the isotope value of the derivatized amino acid standards determined via gas chromatography–isotope ratio mass spectrometry (GC-IRMS). The stable carbon isotope values of the amino acids in the unknown samples were calculated by correcting for the carbon added during derivatization and associated kinetic isotope effects using Equation 1, derived from Docherty et al. (2):

$$\delta^{13}\mathrm{C}_{\mathrm{sample\ aa}} = \frac{\mathrm{n_{aa}} + \mathrm{n_d}}{\mathrm{n_{aa}}}\left(\delta^{13}\mathrm{C}_{\mathrm{deriv\ sample\ aa}} - \delta^{13}\mathrm{C}_{\mathrm{deriv\ standard\ aa}}\right) + \delta^{13}\mathrm{C}_{\mathrm{standard\ aa}} \quad \text{(Equation 1)}$$

where n_{aa} is the number of moles of carbon in the amino acid and n_{d} is the number of moles of carbon added by derivatization. The uncertainty of the calculated amino acid $\delta^{13}C$ values depends on the uncertainty in the EA-IRMS measurement of the underivatized pure standard and the GC-IRMS measurements of derivatized standard and sample, and was calculated using Equation 2 (2):

$$\sigma^2_{\mathrm{sample\ aa}} = \sigma^2_{\mathrm{standard\ aa}} + \sigma^2_{\mathrm{deriv\ standard\ aa}}\left(\frac{\mathrm{n_{aa}}+\mathrm{n_d}}{\mathrm{n_{aa}}}\right)^2 + \sigma^2_{\mathrm{deriv\ sample\ aa}}\left(\frac{\mathrm{n_{aa}}+\mathrm{n_d}}{\mathrm{n_{aa}}}\right)^2 \quad \text{(Equation 2)}$$

where σ is the standard deviation of replicate measurements.

GC-Orbitrap-IRMS standardization and instrument fractionation:

For precise and accurate isotope ratio measurements using GC-Orbitrap-IRMS, sample compositions are determined relative to a known standard. In this study, glycine (≥ 99%, Sigma Aldrich) served as the isotopically characterized standard, while glycine extracted from the Murchison and Bennu samples were analyzed as unknowns. Samples and standards were analyzed in triplicate, with the Sigma Aldrich standard analyzed between sample runs using identical analytical conditions to monitor instrument stability and variability.

Following the framework of Eiler et al. (3), chemically identical compounds exhibit comparable instrumental fractionation behavior. This allows a single isotopically characterized standard of the same compound to reliably transfer measurements onto an absolute isotopic scale. This approach avoids the need for multiple standards spanning a wide isotopic range, which is often required for unknown compounds of different chemistries but is unnecessary for identical molecular structures with similar ionization behavior. Additionally, the analytical error of the GC-Orbitrap-IRMS values exceeds any error that would be introduced by scale compression/non-linearity caused by using a single isotope standard rather than isotopic bracketing, such that additional standards do not improve data quality.

To further minimize analytical bias, standard concentrations were adjusted to closely match the absolute ion intensity of the corresponding sample. This principle of identical treatment reduces differences in injection volume, concentration, or measurement conditions, thereby improving the accuracy and reliability of isotope ratio comparisons by controlling for instrumental mass fractionation (3).

The scan ranges, resolution, and AGC target were optimized to ensure that, under the selected experimental conditions, most of the detected ions originated from the compounds of interest (3). These settings were also chosen to mitigate space-charge effects, a phenomenon in which excessive ion populations in the C-trap or Orbitrap interact, disrupting the stable, harmonic orbits essential for accurate Fourier-transform mass spectrometry (3, 4). Such effects can be reduced by narrowing the mass scan window or lowering the AGC target to limit the number of extraneous ions that might interfere with the measurement of isotope ratios. Additionally, reducing the resolution shortens the amount of time ions spend in the Orbitrap, decreasing the likelihood of ion-ion interactions and orbit instability, while generally enhancing sensitivity and reducing the impact of contaminants on isotope ratio accuracy (4).

A commercial GC-Orbitrap-MS was modified with a peak trapping device to enhance analytical accuracy and precision of isotopic measurements by increasing the width of the chromatographic peak, and thus the number of ions observed per scan in the Orbitrap (see 5, 6). Direct analysis of standards and samples were used to determine the appropriate trapping window based on the retention time of each compound of interest and to rule out any extraneous ions co-eluting with analyte fragments within the mass window of interest.

We were careful to trap only well-resolved and sharp chromatographic peaks to minimize any possible contamination from coeluting compounds. All isotopologue ions showed identical peak shapes and each eluted analyte peak was inspected for extraneous ions. Mass spectra were

scrutinized across the entire untrapped peak to ensure that only the isotopologues' mass-to-charge ratios of interest were present within the target mass windows. While chromatographic separation is often accompanied by carbon isotope fractionation across a GC peak, the elution time differences for an isotopologue pair are on the scale of milliseconds (7, 8), so contaminating compounds will have visibly discernable peak profiles. To avoid any potential influence of chromatographic isotope separation, we were careful to trap the entirety of each peak. Finally, electron ionization (EI) is a relatively energetic method, and to our knowledge, the typically small ion fragments produced from the studied amino acids via EI do not include metastable ions that could further degrade to yield targeted ion fragment masses.

Amino acid intramolecular carbon isotope corrections:

The intramolecular $\delta^{13}C$ values were also corrected for the dilution by carbons from derivatizing reagents present in the fragment of interest. Position-specific $\delta^{13}C$ values were converted to the internationally recognized primary isotope reference scale, Vienna Peedee Belemnite (VPDB), using the following equations for delta values (Equation 3) and error propagation (Equation 4):

$$\delta^{13}C\alpha_{sample,VPDB} = \delta^{13}C\alpha_{sample,\ standard} + \delta^{13}C\alpha_{standard,\ VPDB} + \left(\frac{1}{1000}\right)(\delta^{13}C\alpha_{sample,\ standard})(\delta^{13}C\alpha_{standard,\ VPDB}) \quad \text{(Equation 3)}$$

$$\sigma_{sample,VPDB} = \sqrt{\sigma_{sample,\ standard}^{2} + \sigma_{standard,\ VPDB}^{2} + \left(\frac{1}{1000}\right)^{2} + [(\delta^{13}C\alpha_{standard,\ VPDB} \times \sigma_{sample,\ standard})^{2} + (\delta^{13}C\alpha_{sample,\ standard} \times \sigma_{standard,\ VPDB})^{2}} \quad \text{(Equation 4)}$$

The standardized $\delta^{13}C_{VPDB}$ for the glycine carbon Cα in the fragment was corrected for the dilution by carbons from derivatizing reagents present in the fragment of interest. This correction and the propagated error are found in Equations 5 and 6, respectively:

$$\delta^{13}C\alpha_{corrected} = \delta^{13}C\alpha_{sample,VPDB} \times (nC_{frag}/nC_{gly}) \quad \text{(Equation 5)}$$

$$\sigma_{corrected} = \sigma_{sample,VPDB} \times (nC_{frag}/nC_{gly}) \quad \text{(Equation 6)}$$

where nC_{frag} is the total number of carbons in the measured fragment and nC_{gly} is the number of carbons from glycine in that fragment (e.g., nC_{frag}= 3 and nC_{gly}= 1 for the 126.016 fragment). It is presumed that the external carbon atoms introduced during derivatization had the same isotopic compositions for both the sample and the reference standard, because the sample and standard were derivatized simultaneously under identical conditions with an excess of reagent. Additionally, it is assumed that any instrumental fractionations during measurement affect the sample and standard equally.

The $\delta^{13}C$ value for the carboxylic acid carbon of glycine can then be measured using a mass balance Equation 7 (propagation error showed in Equation 8).

$$\delta^{13}{}_{COOH} = a.\ \delta^{13}C_{molecular\ average} - b \times \delta^{13}C\alpha_{corrected} \quad \text{(Equation 7)}$$

$$\sigma_{COOH} = \sqrt{(a.\ \sigma_{molecular\ average})^{2} + (b.\ \sigma_{corrected})^{2}} \quad \text{(Equation 8)}$$

where a is the total number of carbons in the amino acid (glycine) and b is the number of carbons in the measured fragment.

Table S1: $\delta^{13}C_{COOH}$ and $\delta^{13}C\alpha$ values and standard errors of glycine USGS65 measured with the GC-Orbitrap-IRMS compared to literature values.

	**Glycine USGS65**	
	$\delta^{13}C_{COOH}$	$\delta^{13}C\alpha$
**Orbitrap**	–22.3 ± 4.4	–18.3 ± 4.4
**Literature**	–24.3 ± 0.3[a]	–15.9 ± 0.7[b]

[a]Fry et al. (9); [b]Rasmussen and Hoffman (10)

The intramolecular carbon isotope method for glycine on the GC-Orbitrap-IRMS was validated by analyzing glycine for which the intramolecular isotopic values had been measured by NMR (10) and the ninhydrin reaction (9). Reference material USGS64 was used as the known standard to convert measured values to the VPDB scale. Reference material USGS65 was treated as an unknown sample. Here we show that the values obtained for the carboxylic carbon ($\delta^{13}C_{COOH}$= –22.3 ± 4.4) and the amine carbon ($\delta^{13}C\alpha$= –18.3 ± 4.4) on the Orbitrap for glycine USGS65 are consistent with the literature values obtained for both carbons ($\delta^{13}C_{COOH}$= –24.3 ± 0.3 and $\delta^{13}C\alpha$= –15.9 ± 0.7) (Table S1).

Carbonyl extraction and preparation:

Standards and reagents were purchased from Sigma Aldrich and Fisher Scientific. Ultrapure water (Millipore Direct Q3 UV, 18.2 MΩ, 3 ppb total organic carbon; hereafter referred to as "water"), HPLC grade dichloromethane (DCM), double-distilled 6 M HCl, and O-(2,3,4,5,6-pentafluorobenzyl) hydroxylamine hydrochloride of ≥ 99.0% purity were used. All glassware, ceramics, and tools used for sample preparation and analysis were heated at 500°C overnight before use. All vials were capped with PTFE-lined lids. The powdered samples were flame-sealed in glass ampoules (three ampoules per sample type, 2 mL of water per ampoule) and heated at 100°C for 24 hours to extract SOM. The water supernatants were isolated and split into two equal portions. One half was desalted using cation exchange chromatography. The desalted water wash was subdivided into portions for SOM analyses including, for this work, 37% for carbonyls. The other half of the water extract was dried and acid hydrolyzed (6 M HCl vapor at 150°C for 3 hours) for the analysis of the total (free + HCl-hydrolyzed) amino acid content (11).

Carbonyl derivatization and $\delta^{13}C$ analysis:

The carbonyls were derivatized using an optimized Environmental Protection Agency (EPA) Method #556 with O-(2,3,4,5,6-pentafluorobenzyl) hydroxylamine hydrochloride (PFBHA) (12) then concentrated down to small volumes under a stream of nitrogen and analyzed by gas chromatography–mass spectrometry coupled with isotope ratio mass spectrometry (GC-MS/IRMS). Separation, quantification, and compound-specific carbon stable isotopic analysis of the carbonyl derivatives were carried out on a GC-MS/IRMS instrument suite consisting of a Thermo Trace GC, a Thermo DSQII electron-impact quadruple mass spectrometer, a Thermo GC-C III interface, and a Thermo MAT 253 IRMS. Sample injections were made in splitless mode in

aliquots of 5 μL; splitless mode was used to maximize sensitivity and minimize potential isotopic fractionation during injection. Mass spectra were used to identify and quantify carbonyl compounds in the samples by comparison to reference standards and application of calibration curves. Seven-point external calibration curves were prepared for each individual carbonyl standard. A selected ion mass-to-charge ratio (m/z = 181.0) was used to identify and quantify compounds in the samples. Concentrations were calculated using linear equations derived from the calibration curves of each individual standard. Ten pulses of high-purity reference gas (CO_2) that were calibrated against commercial reference gases with known isotopic ratios were injected into the IRMS for computation of the isotopic ratios of the eluting derivatized carbonyl standards and sample compounds.

Carbonyl CSIA isotope corrections:

To correct for the isotopic contribution from carbon added by the derivatization reagent, $\delta^{13}C$ values were also determined for both derivatized carbonyl standards and for underivatized carbonyl standards using a Costech ECS 4010 combustion elemental analyzer (EA) connected to the IRMS. The final $\delta^{13}C$ values of the carbonyls were calculated using Equation 1 and error was propagated using Equation 2.

Extended Results

Amino acid molecular-averaged $\delta^{13}C$ values:

The molecular-averaged $\delta^{13}C$ values for glycine, ß-alanine, and L-glutamic acid represent replicate analyses ($n \geq 4$) and are therefore reported with a higher degree of confidence (standard deviation ≤ 6‰) than the amino acids for which we only report a single isotope value: D-glutamic acid, D- and L-aspartic acid, and γ-aminobutyric acid.

Table S2. Amino acid molecular-averaged $\delta^{13}C$ values (‰, VPDB) and peak areas (Vs). Samples in black included in data analysis (Table 1). Samples with an asterisk (*) had peak areas that were smaller than the standard peaks and are not included in Table 1 of the manuscript.

Amino Acid	AA $\delta^{13}C$ (‰, VPDB)	Peak Area (Vs)
Glycine	25.3	0.183
	14.4	0.562
	18.7	0.696
	20.0	0.723
	22.7	0.769
	21.7	0.780
ß-alanine	2.8*	0.038*
	11.4*	0.128*
	15.2	0.166
	9.0	0.167
	17.9	0.181
	6.3	0.252

D-aspartic acid	-4.3*	0.066*
	0.5*	0.083*
	-2.7*	0.085*
	3.3*	0.091*
	0.9*	0.098*
	3.1	0.136
D-glutamic acid	14.4*	0.057*
	2.9*	0.069*
	2.2*	0.072*
	-3.0*	0.075*
	11.4*	0.080*
	5.0	0.112
γ-aminobutyric acid	19.3*	0.073*
	10.5*	0.097*
	15.5*	0.098*
	18.9*	0.104*
	16.1*	0.109*
	16.9	0.157
L-aspartic acid	-5.1*	0.066*
	8.2*	0.082*
	2.3*	0.082*
	1.9*	0.090*
	9.2*	0.093*
	3.6	0.126
L-glutamic acid	3.5*	0.060*
	-5.2*	0.075*
	19.6	0.078
	20.7	0.083
	21.2	0.087
	16.8	0.124

Data Availability

Table S3: List of the data products from the Bennu samples analyzed in this study and corresponding DOIs, to be available via AstroMat (https://astromat.org).

Data Type	Sample	DOI	Product Name	Product Type
Amino Acid GC-IRMS	OREX-800107-183	10.60707/0134-b672	20250219_GC-C-IRMS_OREX-800107-183_1_GCCIRMSDataCollection_1.zip	GCCIRMSCollection
		10.60707/8nap-2d05	20250219_GC-C-IRMS_OREX-800107-183_1_GCCIRMSTabularIsotopicValues_1.csv	GCCIRMSTabularIsotopicValues
Amino Acid GC-Orbitrap-IRMS	OREX-800107-183	10.60707/gh1z-1r87	20250509_GC-C-IRMS_KUIAB_OREX-800107-183_1_GCCIRMSOrbitrapCollection_1.zip	GCCIRMSOrbitrapCollection
Carbonyl GC-IRMS	OREX-800107-127	10.60707/40wp-e994	20241024_GC-C-IRMS_GSFC_OREX-800107-127_1_GCCIRMSDataCollection_1.zip	GCCIRMSDataCollection
		10.60707/pynf-qf21	20241029_GC-C-IRMS_GSFC_OREX-800107-127_1_GCCIRMSDataCollection_1.zip	

References:

1. L. T. Corr, R. Berstan, R. P. Evershed, Optimisation of derivatisation procedures for the determination of δ13C values of amino acids by gas chromatography/combustion/isotope ratio mass spectrometry. *Rapid Communications in Mass Spectrometry* **21**, 3759-3771 (2007).
2. G. Docherty, V. Jones, R. P. Evershed, Practical and theoretical considerations in the gas chromatography/combustion/isotope ratio mass spectrometry δ13C analysis of small polyfunctional compounds. *Rapid Communications in Mass Spectrometry* **15**, 730-738 (2001).
3. J. Eiler *et al.*, Analysis of molecular isotopic structures at high precision and accuracy by Orbitrap mass spectrometry. *International Journal of Mass Spectrometry* **422**, 126-142 (2017).
4. A. E. Hofmann *et al.*, Using Orbitrap mass spectrometry to assess the isotopic compositions of individual compounds in mixtures. *International Journal of Mass Spectrometry* **457**, 116410 (2020).
5. O. M. McIntosh *et al.*, Stable Nitrogen Isotope Analysis of Amino Acids by Orbitrap Mass Spectrometry: Application for Extraterrestrial Samples. *Rapid Communications in Mass Spectrometry* **39**, e10127 (2025).
6. Y. J. Sato, A. A. Baczynski, H. Xie, A. Gilbert, K. H. Freeman, Molecular Average and Intramolecular δ13C Measurements of Pyruvic Acid and Acetic Acid Using Gas Chromatography Orbitrap Mass Spectrometry (GC Orbitrap-MS). *Analytical Chemistry* **97**, 19047-19056 (2025).
7. K. H. Freeman, J. M. Hayes, J.-M. Trendel, P. Albrecht, Evidence from carbon isotope measurements for diverse origins of sedimentary hydrocarbons. *Nature* **343**, 254-256 (1990).
8. M. P. Ricci, D. A. Merritt, K. H. Freeman, J. M. Hayes, Acquisition and processing of data for isotope-ratio-monitoring mass spectrometry. *Organic Geochemistry* **21**, 561-571 (1994).
9. B. Fry, J. F. Carter, K. Yamada, N. Yoshida, D. Juchelka, Position-specific 13C/12C analysis of amino acid carboxyl groups – automated flow-injection analysis based on reaction with ninhydrin. *Rapid Communications in Mass Spectrometry* **32**, 992-1000 (2018).
10. C. Rasmussen, D. W. Hoffman, Novel Nuclear Magnetic Resonance Method for Position-Specific Carbon Isotope Analysis of Organic Molecules with Significant Impurities. *Analytical Chemistry* **94**, 15124-15131 (2022).
11. D. S. Lauretta, H. C. Connolly Jr, J. N. Grossman, A. T. Polit, OSIRIS-REx Sample Analysis Plan--Revision 3.0. *arXiv preprint arXiv:2308.11794* (2023).
12. D. N. Simkus, J. C. Aponte, R. W. Hilts, J. E. Elsila, C. D. K. Herd, Compound-specific carbon isotope compositions of aldehydes and ketones in the Murchison meteorite. *Meteoritics & Planetary Science* **54**, 142-156 (2019).

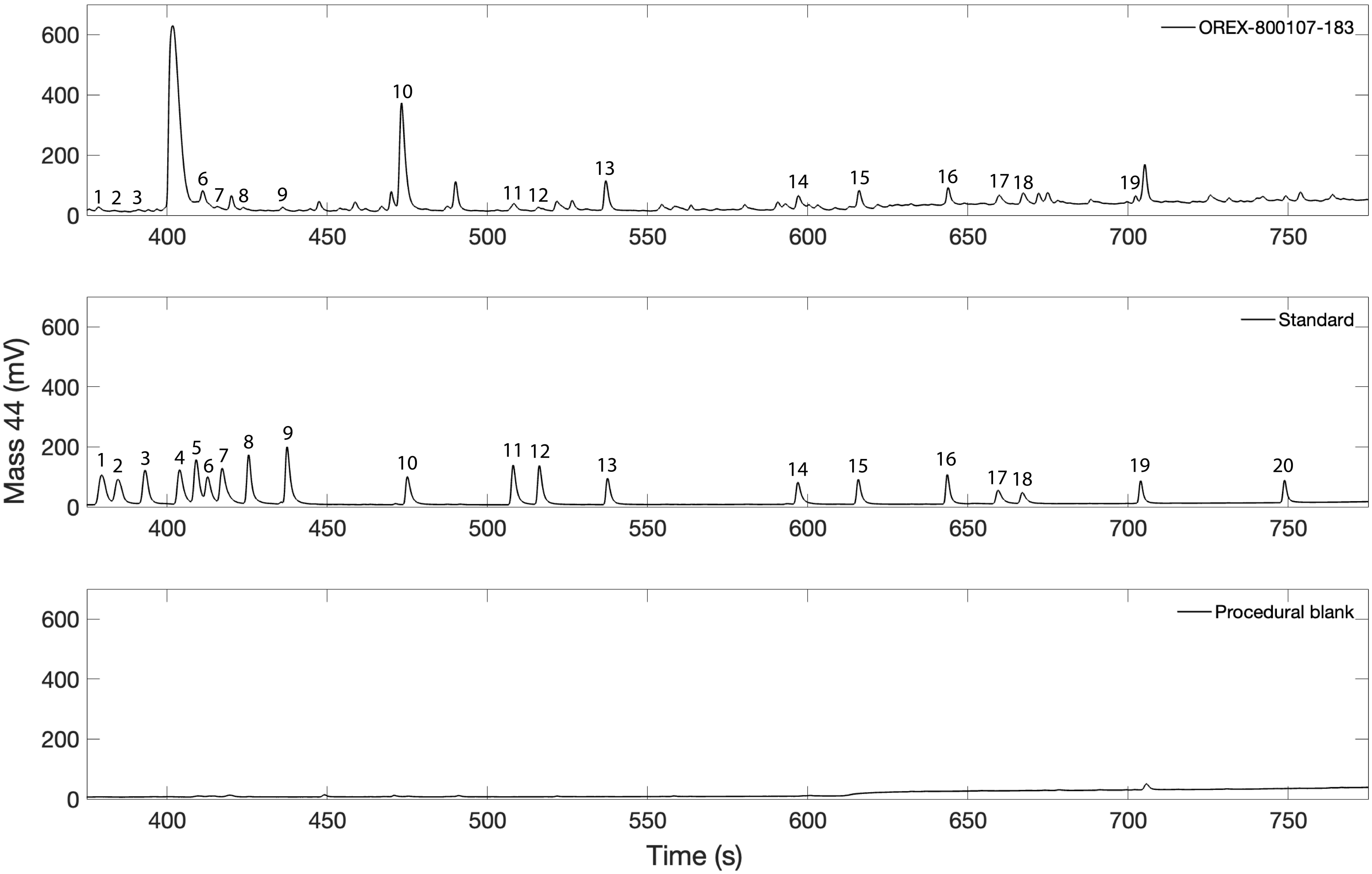

Supplementary Figure S1. Representative pico-CSIA chromatograms for OREX-800107-183, amino acid standard mixture, and procedural blank. Amino acids are as follows: (1) α-aminoisobutyric acid, (2) L-isovaline, (3) D-isovaline, (4) D-alanine, (5) D-valine, (6) L-alanine, (7) D-α-aminobutyric acid, (8) L-valine, (9) L-α-aminobutyric acid, (10) glycine, (11) D-ß-aminoisobutyric acid, (12) L-ß-aminoisobutyric acid, (13) ß-alanine, (14) D-aspartic acid, (15) L-aspartic acid, (16) γ-aminobutyric acid, (17) D-glutamic acid, (18) L-glutamic acid, (19) δ-amino-n-valeric acid, and (20) ε-amino-n-caproic acid. 19 of the 20 amino acids in the standard mixture were identified in OREX-800107-183. OREX-800107183 has a large, unidentified peak beginning at ~400s. This peak obscures both D-alanine and D-valine. Peaks that are unlabeled are unidentified.

Strecker-cyanohydrin synthetic pathway:

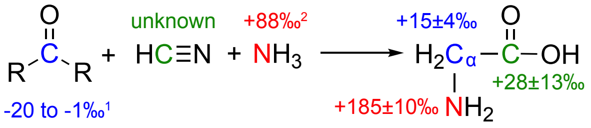

Plausible, but unlikely due to carbon and nitrogen isotopic disagreement.

Reductive amination:

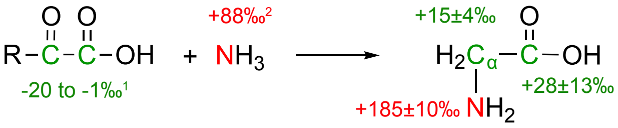

Plausible, but unlikely due to carbon and nitrogen isotopic disagreement.

Ammonia-involved formose-type reaction:

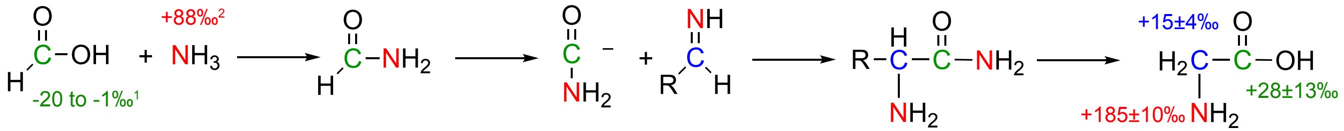

Plausible, but unlikely due to carbon and nitrogen isotopic disagreement.

Michael addition:

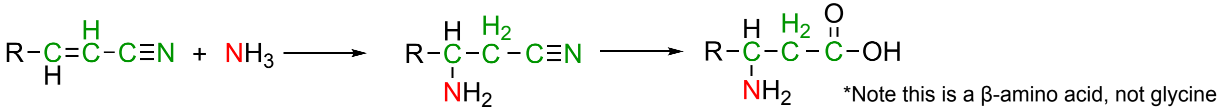

Not possible; does not produce α-amino acids.

CO_2 addition to amines:

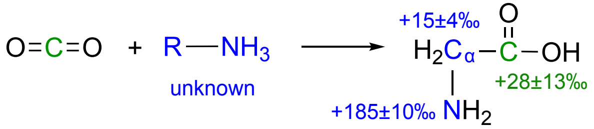

Plausible, but unlikely due to precursor amines and CO_2 needing to have similar carbon isotope values, which disagrees with theoretical predictions.

Modified radical-radical synthetic pathway:

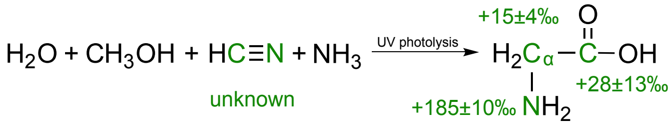

Plausible and most likely mechanism due to HCN contributing both carbon atoms to glycine. Future carbon and nitrogen isotopic measurements of Bennu HCN needed to test hypothesis.

Supplementary Figure S2. Amino Acid formation mechanisms and predictions for Bennu. Modified from Elsila et al., 2012. Values for precursor compounds from this study (Table 2) [1]; Foustoukos et al. (2025) [2].
